# AF1q Mediates Tumor Progression in Colorectal Cancer by Regulating AKT Signaling

**DOI:** 10.3390/ijms18050987

**Published:** 2017-05-05

**Authors:** Jingwei Hu, Guodong Li, Liang Liu, Yatao Wang, Xiaolan Li, Jianping Gong

**Affiliations:** Cancer Research Institute, Tongji Hospital, Huazhong University of Science and Technology, 1095 Jiefang Ave., Wuhan 430030, China; hjwmed@sina.com (J.H.); gdli1986@163.com (G.L.); liuliang1108@126.com (L.L.); wyt19920427@126.com (Y.W.); xlli@tjh.tjmu.edu.cn (X.L.)

**Keywords:** colorectal cancer, *AF1q* gene, epithelial-mesenchymal transition, metastasis

## Abstract

The up-regulation of *ALL1*-fused gene from chromosome 1q (AF1q) is commonly seen in aggressive hematologic malignancies as well as in several solid tumor tissues. However, its expression and intrinsic function in human colorectal cancer (CRC) remains largely undefined. To explore the role of AF1q in human CRC progression, AF1q expression was analyzed in human CRC tissue samples and CRC cell lines. Clinical specimens revealed that AF1q was up-regulated in human CRC tissues, and that this up-regulation was associated with tumor metastasis and late tumor, lymph node, metastasis (TNM) stage. *AF1q* knockdown by shRNA inhibited tumor cell proliferation, migration, invasion, and epithelial-mesenchymal transition in vitro, as well as tumorigenesis and liver metastasis in vivo, whereas these effects were reversed following *AF1q* overexpression. These AF1q-mediated effects were modulated by the protein kinase B (AKT) signaling pathway, and inhibition of AKT signaling attenuated AF1q-induced tumor promotion. Thus, AF1q contributes to CRC tumorigenesis and progression through the activation of the AKT signaling pathway. AF1q might therefore serve as a promising new target in the treatment of CRC.

## 1. Introduction

Colorectal cancer (CRC) is the third most common human malignant neoplasm worldwide, and prognosis varies significantly between early- and late-stage diseases [1,2]. The five-year overall survival (OS) rate of stage II CRC patients is as high as 80% following surgery, but this drops to 40–60% for stage III CRC patients, who display evidence of tumor invasion and regional lymph node metastasis [3]. A clearer understanding of the mechanisms underlying CRC development and metastasis may allow the development of better diagnostic and therapeutic strategies.

*ALL1*-fused gene from chromosome 1q (*AF1q*), also known as *MLLT11*, is located on chromosome 1q21 and encodes a 9 kDa, 90-amino-acid protein. It was first identified in an acute myeloid leukemia patient who carried the *t*(1;11)(q21;q23) chromosomal translocation [4]. AF1q overexpression has been observed in aggressive hematologic malignancies as well as in several solid neoplasms, including breast carcinoma, thyroid neoplasm, testicular neoplasm, and neuroblastoma [5,6,7,8,9]. AF1q is also significantly up-regulated during neuronal differentiation and development [10]. MicroRNA 29b binds to the *AF1q* 3′-untranslated region to inhibit its expression [11]. Additionally, promoter analyses have suggested that the *AF1q* gene and the small protein effector of Cdc42 (*SPEC1*) gene share a promoter region, but these genes are not coordinately regulated [12]. AF1q is considered an adverse prognostic factor in acute monocytic leukemia and myelodysplastic syndrome [13,14]; AF1q binding to the transcription factor T-cell-factor-7 may result in activation of the wingless-type MMTV integration site family (Wnt) signaling pathway, thus promoting tumor dissemination [8], and AF1q up-regulation is reportedly correlated with distant metastasis in lung and breast cancers [15,16,17]. Conversely, AF1q has also been reported to influence the pro-apoptotic effects mediated by doxorubicin or retinoic acid [5,6]. Therefore, the precise function and intrinsic mechanisms of AF1q warrant further investigation.

To date, there have been no reports of the association between AF1q and CRC. Therefore, in this study, we analyzed AF1q expression in clinical CRC specimens, and explored the role of AF1q in the biological behavior of CRC cells, both in vitro and in vivo. Our data provide evidence of AF1q influencing both the epithelial–mesenchymal transition (EMT) and the AKT signaling pathway.

## 2. Results

### 2.1. AF1q Was Upregulated in Human CRC Specimens and Was Indicative of Poor Prognosis

**Figure 1:**
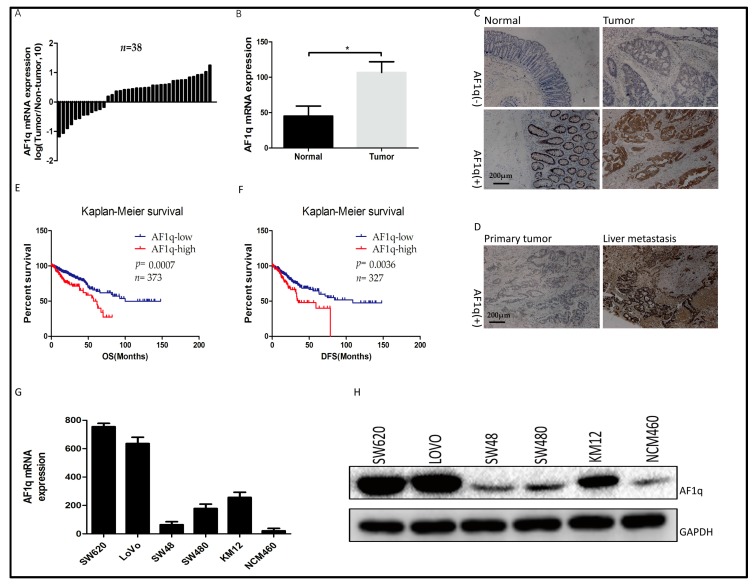
Expression analyses of AF1q in colorectal cancer (CRC) tissue specimens and cell lines. (**A**) qPCR analyses of AF1q mRNA expression level in 38 CRC tissues and paired normal tissues; (**B**) Histogram showing the average AF1q mRNA expression level in 38 CRC and paired normal tissues; (**C**,**D**) Representative immunostaining images showing AF1q expression in normal tissues, CRC tissues, and liver metastasis tissues (magnification: 100×). Scale bars: 200 μm; (**E**,**F**) Kaplan-Meier overall (OS) and disease free (DFS) survival analysis for CRC patients classified as having high or low levels of AF1q mRNA expression at the primary tumor site. For this classification, RNASeq data was downloaded from TCGA website [18,19]; (**G**) qPCR analyses of AF1q mRNA expression in five CRC cell lines (SW620, LoVo, SW48, SW480, and KM12) and one normal intestinal epithelial cell line (NCM460); (**H**) Western blot analyses of AF1q protein in the cell lines described in (**G**). Data are presented as the mean ± standard deviation, and are the average of three independent experiments. * *p* < 0.05.

To explore the biological role of AF1q in human CRC progression, AF1q expression was analyzed in clinical CRC tissue samples. Firstly, AF1q mRNA expression was examined by quantitative RT-PCR using 38 pairs of CRC specimens and patient-matched normal tissues, and was found to be higher in the tumor tissues than in normal colorectal tissues (Figure 1A,B). Next, AF1q protein expression was examined by immunohistochemical staining (IHC), using 96 pairs of CRC specimens and their matched normal tissues. Among the 96 pairs of samples, 54 CRC tissues (56.3%) and 28 normal tissues (29.2%) were found to be positive for AF1q expression (*p* < 0.05), with the AF1q protein being primarily located in the cell cytoplasm. Representative AF1q immunostaining images for normal, CRC, and metastatic liver tissues are shown in Figure 1C,D. AF1q up-regulation was significantly associated with lymph node metastasis (*p* = 0.0159) and late tumor, lymph node, metastasis (TNM) stage (*p* = 0.018; Table 1).

Bioinformatic analysis was performed to examine the relationship between AF1q and CRC patient survival outcomes. For this analysis, level 3 HiSeq RNA-Seq data for 380 cancer samples were downloaded from The Cancer Genome Atlas (TCGA) website [18,19]. Then, the R package ‘survival’ was used to analyze the relationship between AF1q expression and survival, using data from the TCGA and other CRC cohorts. The results showed that AF1q up-regulation was indicative of both poor overall survival (Figure 1E) and poor disease-free survival (Figure 1F). Following this result, AF1q expression was evaluated in five CRC cell lines (SW480, SW48, KM12, SW620, and LoVo) and one normal intestinal epithelial cell line (NCM460). As shown in Figure 1G,H, AF1q expression at both the mRNA and the protein level was higher in the five CRC cell lines than in NCM460 cells. These results suggest that AF1q is overexpressed in CRC, and that this increased expression is predictive of poor prognosis in CRC patients.

### 2.2. AF1q Promotes CRC Cell Proliferation In Vitro

**Figure 2:**
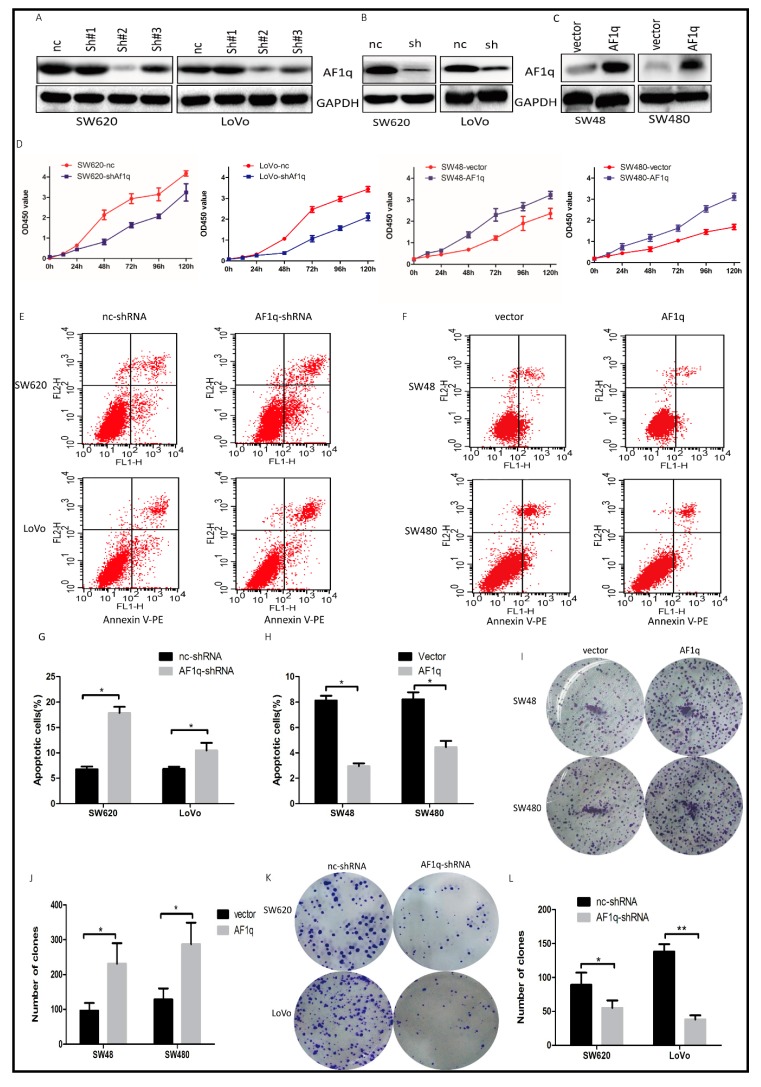
The effect of AF1q on CRC cell proliferation. (**A**) Western blot showing the effect of transiently transfected shRNAs sh#1, sh#2, and sh#3 on AF1q expression in SW620 and LoVo cells. Also shown is the negative control (nc) shRNA; (**B**) Western blot analysis of AF1q expression in SW620 and LoVo cells stably transfected with AF1q-shRNA (sh) or negative control (nc); (**C**) Western blot analysis of AF1q expression in SW48 and SW480 cells transfected with AF1q-expressing plasmid (AF1q) or empty vector control (vector); (**D**) The effect of *AF1q* knockdown (SW620 and LoVo) or overexpression (SW48 and SW480) on cell proliferation, as assessed by CCK8 assay; (**E**,**G**) The effect of *AF1q* knockdown in SW620 and LoVo cells on cell apoptosis, as determined using flow cytometry. Shown are representative flow cytometric data (**E**), and a summary of apoptosis in cells with or without *AF1q* knockdown (**G**); (**F**,**H**) The effect of *AF1q* overexpression in SW48 and SW480 cells on cell apoptosis; (**I**,**J**) The effect of *AF1q* overexpression on colony formation ability after 2 weeks, showing representative colony forming assays (**I**) and summary data (**J**) for each cell line, with and without *AF1q* overexpression; (**K**,**L**) The effect of *AF1q* knockdown on colony formation ability. Data are presented as the mean ± standard deviation of three independent samples. * *p* < 0.05, ** *p* < 0.01.

Having established a potential link between AF1q overexpression and CRC progression, we investigated whether *AF1q* knockdown affected cell proliferation in CRC. Three *AF1q* shRNAs (sh#1, sh#2, and sh#3) were designed and transfected into the CRC cell lines with the highest AF1q expression, SW620 and LoVo. Of these, sh#2 silenced AF1q expression with the greatest efficiency (Figure 2A), and was used to establish SW620/AF1q-shRNA and LoVo/AF1q-shRNA cell lines, which exhibited stable AF1q down-regulation (Figure 2B). Meanwhile, an AF1q-expressing vector was transfected into the CRC cell lines with low basal AF1q expression levels, SW48 and SW480, to examine AF1q up-regulation in CRC cells (Figure 2C). Our results showed that AF1q down-regulation significantly inhibited CRC cell proliferation, while AF1q up-regulation had the opposite effect (Figure 2D).

Next, we examined cell apoptosis by flow cytometry, using Annexin V/PI to stain *AF1q*-knockdown cells. The number of apoptotic cells was significantly higher in the SW620/AF1q-shRNA and LoVo/AF1q-shRNA cells than in the SW620/nc-shRNA and LoVo/nc-shRNA cells (Figure 2E,G), indicating that *AF1q* knockdown promotes apoptosis. Meanwhile, *AF1q* overexpression in SW48 and SW480 cells showed the opposite effect (Figure 2F,H). Furthermore, using a colony-formation assay, we found that both SW48/AF1q and SW480/AF1q cells had enhanced colony-formation ability compared with their respective controls (Figure 2I,J), while SW620/AF1q-shRNA and LoVo/AF1q-shRNA cells had impaired colony-formation ability (Figure 2K,L). Taken together, these results indicate that AF1q promotes CRC cell proliferation in vitro.

### 2.3. AF1q Promotes CRC Cell Migration, Invasion, and EMT In Vitro

**Figure 3:**
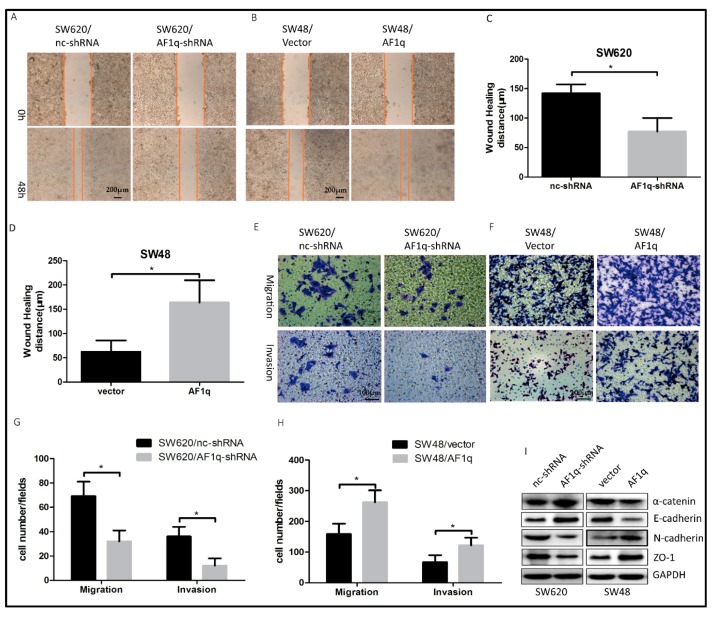
AF1q promotes migration and invasion of CRC cells in vitro. (**A**,**B**) Representative wound healing assays showing that *AF1q* knockdown reduced the invasive potential of SW620 cells (**A**), while overexpression of *AF1q* promoted the invasive potential of SW48 cells (**B**) (magnification: 100×). Scale bars: 200 μm; (**C**,**D**) Histograms showing the average wound healing distance (μm) for the cells shown in (**A**,**B**), respectively; (**E**,**F**) Representative transwell assays showing that *AF1q* knockdown inhibited SW620 cell invasion and migration (**E**), while *AF1q* overexpression promoted SW48 cell invasion and migration (**F**) (magnification: 200×). Scale bars: 100 μm; (**G**,**H**) Histograms showing the average level of cell migration, from the data shown in (**E**,**F**), respectively; Statistical analysis was performed using three microscopic fields selected at random; (**I**) Western blot analysis of the indicated epithelial–mesenchymal (EMT) markers (α-catenin, E-cadherin, N-cadherin and ZO-1) following *AF1q* knockdown or overexpression in SW620 cells or SW48 cells, respectively. Data are represented as the mean ± standard deviation of three independent experiments. * *p* < 0.05.

The metastatic potential of cancer cells is associated with enhanced cell mobility [20]. Next, we measured CRC cell migration and invasion ability following *AF1q* knockdown or overexpression. We found that *AF1q* down-regulation suppressed CRC cell wound-healing ability (Figure 3A,C), while, conversely, *AF1q* up-regulation promoted wound-healing in these cells (Figure 3B,D). Furthermore, *AF1q* knockdown significantly inhibited CRC cell migration and invasion ability (Figure 3E,G), while the opposite effects were induced by *AF1q* overexpression (Figure 3F,H). EMT is a crucial process in tumor development, during which tumor cells acquire enhanced migration and invasion abilities [21]. To further investigate the intrinsic mechanisms by which AF1q promotes CRC, we examined the relationship between AF1q status and EMT in CRC cells. *AF1q* knockdown increased the expression of epithelial cell markers (α-catenin and E-cadherin), and decreased the expression of mesenchymal cell markers (N-cadherin and ZO-1), while, conversely, *AF1q* overexpression significantly promoted the EMT phenotype (Figure 3I). Taken together, these results suggest that AF1q promotes CRC cell migration and invasion via the induction of EMT.

### 2.4. AF1q-Induced CRC Tumor Promotion Is Mediated by Activation of the AKT Signaling Pathway

**Figure 4:**
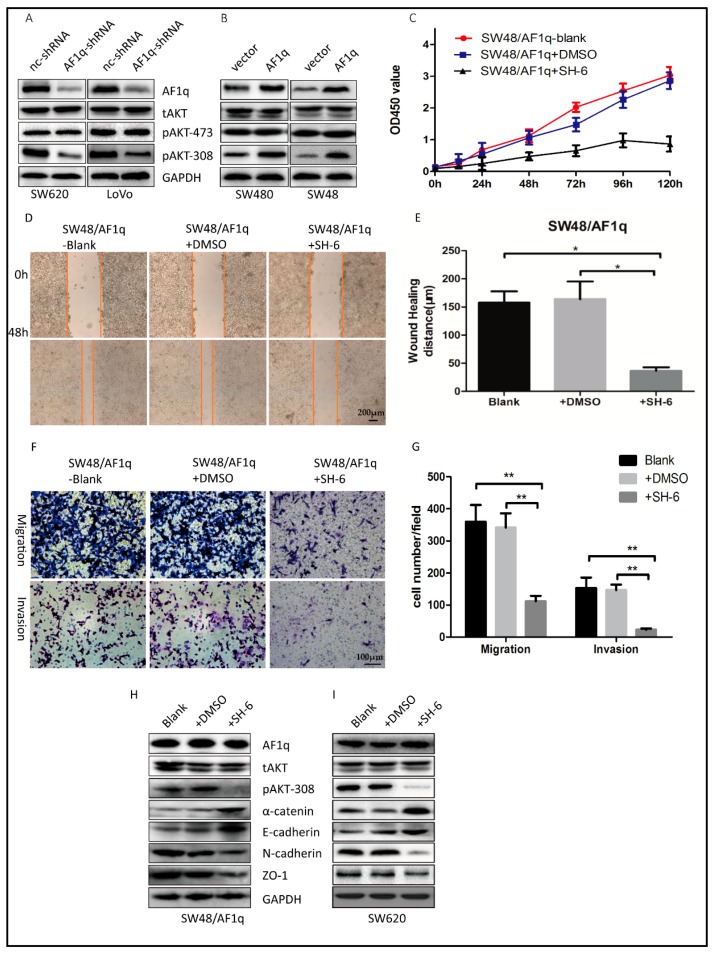
AF1q promotes CRC cell migration, invasion, and EMT via the protein kinase B (AKT) signaling pathway. (**A**,**B**) Western blot analysis of tAKT, pAKT-473, and pAKT-308 following either knockdown (**A**) or overexpression (**B**) of *AF1q*; (**C**) SW48/AF1q cell proliferation in the presence and absence of the AKT phosphorylation inhibitor SH-6, as determined by CCK8 assay; (**D**) Representative wound healing assays for SW48/AF1q cells with and without SH-6 (magnification: 100×). Scale bar: 200 μm; (**E**) Summary of the data shown in (**D**); (**F**) Representative cell migration and invasion assays for SW48/AF1q cells with or without SH-6 (magnification: 200×). Scale bar: 100 μm; (**G**) Summary of the data shown in (**F**); (**H**,**I**) Western blot showing the effect of SH-6 on expression of the EMT markers α-catenin, E-cadherin, N-cadherin, and ZO-1 in SW48/AF1q cells (**H**) and in SW620 cells (**I**). Data are presented as the mean ± standard deviation, and are representative of three independent experiments. * *p* < 0.05, ** *p* < 0.01.

AF1q reportedly up-regulates the signaling of platelet-derived growth factor receptor (PDGFR), which is functionally involved in AKT phosphorylation [17,22]. In order to characterize the mechanisms by which AF1q promotes CRC tumorigenesis, we investigated the effect of AF1q on AKT phosphorylation in CRC cells. To this end, protein expression of total AKT (tAKT), AKT phosphorylated at serine 473 (pAKT-473), and AKT phosphorylated at threonine 308 (pAKT-308) were examined in both *AF1q*-knockdown and *AF1q*-overexpressing CRC cells by Western blot. *AF1q* knockdown decreased pAKT-308 expression, whereas *AF1q* overexpression had the opposite effect. However, AF1q expression had no effect on tAKT or pAKT-473 expression (Figure 4A,B). This suggests that AF1q is involved in AKT phosphorylation at Thr308 specifically.

Next, we treated SW48/AF1q cells with the selective AKT inhibitor SH-6. We found that the cell proliferation, wound-healing, migration, and invasion capacity was reversed after inhibition of AKT with 10 μM SH-6 (Figure 4C–G). Furthermore, SH-6 reversed the EMT and inhibited pAKT-308 expression in SW48/AF1q cells (Figure 4H). In SW620 cells, which have high expression level of AF1q, SH-6 also reversed the EMT and inhibited pAKT-308 expression (Figure 4I). Together, AKT inhibition with SH-6 showed similar effects with *AF1q* knockdown. These results are compatible with the idea that AF1q regulates CRC cell proliferation, migration, invasion, and EMT induction by activating the AKT signaling pathway.

### 2.5. AF1q Down-Regulation Inhibits CRC Cell Proliferation and Metastasis In Vivo

**Figure 5:**
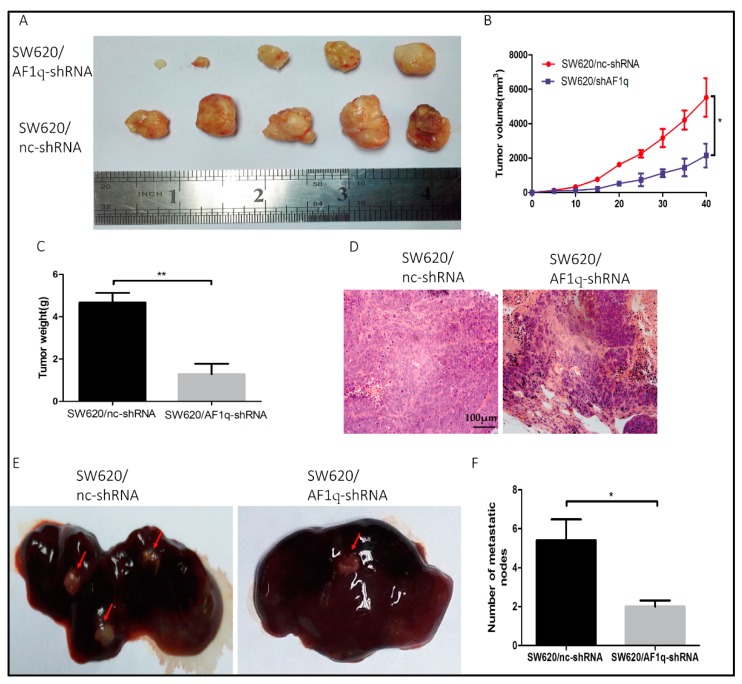
AF1q down-regulation inhibits the CRC cell proliferation and liver metastasis in vivo. (**A**) Gross observation of tumor mass. Tumor size was measured every five days and all mice were euthanized 40 days after injection with CRC cells; (**B**) Xenograft tumor growth curves; (**C**) Xenograft tumor weight (*n* = 5); (**D**) Representative hematoxylin-eosin staining of xenograft tumors from both mouse models (magnification: 200×). Scale bar: 100 μm; (**E**) Metastatic lesions in the livers of nude mice injected in the subcapsular splenic region with SW620/nc-shRNA or SW620/AF1q-shRNA cells (2 × 10^6^ cells/mouse). Livers were examined 6 weeks after injection; Red arrows indicate liver metastatic lesions. (**F**) Summary of the number of liver metastatic nodules in each of the groups described in (**E**). Data are represented as the mean ± standard deviation of three independent experiments. * *p* < 0.05, ** *p* < 0.01.

Having thoroughly examined the effects of AF1q in vitro, we further explored the effects of AF1q in vivo using a xenograft model, by injecting SW620/AF1q-shRNA cells or SW620/nc-shRNA cells subcutaneously into nude mice. The tumors originating from SW620/AF1q-shRNA cells were significantly smaller than tumors from the control SW620/nc-shRNA cells (Figure 5A,C,D). Furthermore, the tumors formed from SW620/AF1q-shRNA cells proliferated more slowly than those originating from SW620/nc-shRNA cells (Figure 5B). Next, to investigate CRC liver metastasis, SW620/nc-shRNA and SW620/AF1q-shRNA clonal cells were injected into the subcapsular splenic region in nude mice. Four weeks later, the mice were euthanized and their livers were investigated. Overall, 80% (4/5) of the SW620/nc-shRNA mice exhibited liver metastases, compared with 40% (2/5) of SW620/AF1q-shRNA mice (Figure 5E). Similarly, the number of liver metastatic nodules significantly decreased when AF1q was down-regulated (Figure 5F). Taken together, these data show that AF1q down-regulation inhibits CRC tumor growth and liver metastasis in vivo.

## 3. Discussion

Tumor invasion and metastasis are the primary causes of death in cancer patients, with tumor cell invasion being a key step in tumor progression [23]. While AF1q has been described in malignancies such as leukemia and breast carcinoma, its role in CRC progression remained unclear. In this study, we therefore explored the biological function of AF1q in CRC using clinical specimens and various CRC cell lines. We found that AF1q expression level in CRC cell lines was higher than that in normal intestinal epithelial cell lines. SW48 and SW480 cell lines were derived from primary tumors, and SW620 and LoVo derived from metastatic tumors [24,25]. Expression levels of AF1q in SW48 and SW480 cells were lower than in the two cell lines derived from metastatic tumors, which suggest that AF1q may play an important role in CRC development.

Further supporting this assumption, stable cell lines with *AF1q* overexpression or knockdown were generated. AF1q up-regulation in CRC cells was associated with enhanced proliferation, migration, and invasion in vitro and was found to promote tumor growth and liver metastasis in vivo. Furthermore, AF1q was up-regulated in clinical CRC specimens, and experiments using IHC demonstrated that high AF1q expression was associated with advanced TNM stage and local lymphatic metastasis. More importantly, high AF1q expression predicted poor overall survival and poor disease-free survival. Taken together, our data strongly suggest that AF1q contributes to CRC invasion and metastasis.

EMT plays an important role in tumor progression, through which cancer cells enhance their motility, invasiveness, and metastatic potential [26,27], and the EMT phenotype change is thought to be correlated with cancer grade and TNM stage [28]. Despite many studies into EMT, the intrinsic molecular mechanisms remain unclear. Presently, more than 11 pathways, including the PTEN/AKT/HIF-1α, TGFβ/Wnt, mTOR/NF-κB, and HGF/c-Met pathways, have been shown to be associated with EMT in CRC cells [29]. Furthermore, aberrant AKT pathway activation is thought to be a key step in EMT progression [29,30]. In this study we have shown that AF1q affects the EMT pathway; *AF1q* overexpression resulted in reduced expression of the epithelial markers α-catenin and E-cadherin, while *AF1q* down-regulation promoted expression of the mesenchymal markers N-cadherin and ZO-1. This result demonstrated that AF1q is associated with EMT.

Recently, the CRC Subtyping Consortium have proposed four consensus molecular subtypes (CMSs) of CRC with distinguishing features: CMS1 (microsatellite instability immune), hypermutated, microsatellite unstable and strong immune activation; CMS2 (canonical), epithelial, marked Wnt and V-Myc avian myelocytomatosis viral oncogene homolog signaling activation; CMS3 (metabolic), epithelial and evident metabolic dysregulation; and CMS4 (mesenchymal), prominent transforming growth factor-β activation, stromal invasion and angiogenesis [31]. Subtype CMS4 tumors showed clear up-regulation of genes implicated in EMT, and are related to advanced stages (III and IV) and poor prognosis. AF1q up-regulated CRC tumors displayed similar properties with subtype CMS4 tumors. In this sense, AF1q may be a potential candidate marker for subtype CMS4 tumors and EMT.

AKT is a principal downstream target of the PI3K signaling pathway, and is a pivotal regulator of cell proliferation, apoptosis, invasion, cell cycle control, and EMT [32]; furthermore, AKT activation is associated with poor prognosis in CRC [33,34]. AKT is normally inactive, but can be activated by phosphorylation at Thr308 or Ser473 [35]. Phosphorylation of both of these residues results in maximal AKT activity, but several studies have shown that phosphorylation of either residue can occur independently [35,36]. AKT phosphorylation at Thr308 in lung cancer and acute myeloid leukemia has been shown to be associated with poorer prognosis [36,37]. The relationship between AKT and STAT3 has been reported previously [38,39]. However, while *AF1q* up-regulation may result in the activation of PDGFR/STAT3 signaling [17], a functional relationship between AF1q and AKT has not been reported. Consistent with these studies, our findings revealed that *AF1q* up-regulation resulted in activation of the AKT pathway, leading to enhanced CRC tumor progression and invasion. These effects were associated with AKT phosphorylation at Thr308, but not at Ser473.

Several analogs of phosphatidyl inositol phosphates (PIAs) have been designed to inhibit AKT signaling for cancer treatment [40,41,42]. SH-6 is one of them. PIAs can inhibit AKT and selectively induce apoptosis in cell lines with high levels of AKT expression, without affecting upstream kinases, such as PI3k or phosphoinositide-dependent kinase-1 [41]. Meanwhile, PIAs can also activate the stress kinase, p38α, through MKK3/6-independent and -dependent mechanisms [40]. Further to these observations, we showed that the selective AKT inhibitor SH-6 significantly inhibited CRC tumor progression, and reversed the EMT phenotype in AF1q-overexpressing cells. This means that the function of AF1q can be reversed by AKT inhibition. Previously, the targeted inhibition of AKT activation has shown promise in the treatment of CRC [43]. Several AKT inhibitors have been developed, and some ongoing clinical studies have shown promising outcomes, for instance with MK2206 [44,45]. While our study did not use a recently developed AKT inhibitor, our results are consistent with those reported in clinical trials, and suggest that AF1q expression levels should be considered before beginning AKT inhibitor treatment.

## 4. Materials and Methods

### 4.1. Ethics Statement

The present study was approved by the Tongji Hospital research committee, at the Huazhong University of Science and Technology, China (No. TJ-rc2015362; Date: 24 March 2015). All procedures involving human specimens and animal experiments were approved by the Huazhong University of Science and Technology Ethics Committee (No. TJ-c20150803; Date: 3 August 2015). All recruited patients provided written informed consent.

### 4.2. Cell Culture

The human CRC cell lines SW620, LoVo, SW48, SW480 and KM12, and the normal intestinal epithelial cell line NCM460, were purchased from the Cell Bank of the Chinese Academy of Sciences (Shanghai, China). All cell lines were tested for mycoplasma contamination, and were cultured at 37 °C with 5% CO_2_ in Dulbecco’s modified Eagle’s medium with 10% fetal bovine serum (FBS). SH-6 was purchased from Santa Cruz Biotechnology (Santa Cruz, CA, USA). When co-cultured with SH-6, the cells were first serum staved for 24 h, then exposed to 10 μM SH-6 in serum-containing Dulbecco’s modified Eagle’s medium for 48 h before next step experiments [46].

### 4.3. Tissue Samples and Histological Examination

CRC tissues, normal tissues, and liver metastasis specimens were collected between January 2012 and December 2015 from 96 CRC patients who received surgery at the Gastrointestinal Surgery Department at Tongji Hospital, Huazhong University of Science and Technology, Wuhan, China. None of these patients had received previous radiotherapy or chemotherapy. Pathological diagnosis and TNM staging were confirmed by two experienced pathologists. Fresh tissues were stored in liquid nitrogen before RNA extraction.

### 4.4. Immunohistochemistry

Surgical samples were fixed in 4% paraformaldehyde for 24 h, embedded in paraffin, and sliced into 4 μm-thick sections according to standard protocols. Tissues were then deparaffinized, rehydrated, and boiled with 0.01 M citrate buffer (pH 6.0). Endogenous peroxide activity was blocked with 3% H_2_O_2_, and tissues were stained using an anti-AF1q primary antibody (Abcam, Cambridge, MA, USA).

### 4.5. RNA Extraction and qRT-PCR

RNA was isolated using TRIzol^®^ reagent (Invitrogen, Carlsbad, CA, USA), according to the manufacturer’s protocol. Reverse transcription to generate cDNA was then performed using a RevertAid™ First Strand cDNA Synthesis kit (Fermentas, Hamilton, ON, Canada), according to the manufacturer’s protocol. qRT-PCR assays were carried out using SYBR reagent (Invitrogen) and AF1q and GAPDH primers obtained from RiboBio (Guangzhou, China).

### 4.6. Vector Construction and Transfection

Three *AF1q* shRNAs, a negative control shRNA, and an *AF1q* overexpression vector and corresponding control vector were designed and synthesized by Vigenebio (Jinan, China). Stably transfected *AF1q*-overexpressing and *AF1q* knockdown CRC cells were selected with 1 μg/mL puromycin. Knockdown or overexpression of AF1q was confirmed by Western blot.

### 4.7. Cell Proliferation Assay

Cells were plated in 96-well plates at a density of 5000 cells/well, and cultured at 37 °C with 5% CO_2_. Cell proliferation was then measured using a CCK-8 kit (Dojindo, Kumamoto, Japan) according to the manufacturer’s instructions. Proliferation was monitored by measuring the optical density at 450 nm every 24 h.

### 4.8. Cell Apoptosis Assay

In apoptosis assays, cells were harvested and labeled with Annexin V and propidium iodide (Merck-Millipore, Darmstadt, Germany) according to manufacturer’s instructions. Apoptotic cells were then identified and quantified using a Flow Cytometer (BD Biosciences, San Jose, CA, USA). 

### 4.9. Western Blot Assay and Antibodies

For western blotting, cells were lysed on ice in NP-40 lysis buffer supplemented with a protease inhibitor cocktail (Roche Applied Science, Indianapolis, IN, USA). An appropriate volume of loading buffer was added to samples, and these were boiled for 5 min. Proteins were then separated on a 10–12% SDS-PAGE gradient gel, and electrophoretically transferred to a polyvinylidene difluoride membrane. Proteins were detected using specific primary antibodies from the following suppliers: anti-AF1q (Abcam); anti-α-catenin, anti-E-cadherin, anti-N-cadherin, and anti-ZO-1 (BD Biosciences); anti-p-AKT308, anti-tAKT, anti-pAKT-473, and anti-GAPDH (Santa Cruz).

### 4.10. Wound-Healing Assays

CRC cells were cultured as a monolayer to 100% confluence in 6-well culture plates, then a scratch was inflicted with a sterile pipette tip. Images were taken 0 and 48 h after infliction of the scratch, and the results were analyzed with NIH Image software version 1.55. For each cell type, three random microscopic fields were selected for statistical analysis.

### 4.11. Migration and Invasion Assays

Transwell plates (BD Biosciences) were used to analyze cellular migration ability, and transwell plates coated with Matrigel (BD Biosciences) were used in invasion assays. In both cases, cells in serum-free DMEM were added to the upper chamber, and DMEM with 20% FBS was applied to the lower chamber. After 48 h, residual cells were removed from the upper chamber. Cells were then fixed with methanol, and stained with crystal violet. For each cell type, three random microscopic fields were selected for statistical analysis.

### 4.12. Xenograft Tumor Growth and Metastasis Assays

BALB/C-nu/nu nude mice were purchased from the Shanghai Laboratory Animal Center (Shanghai, China). SW620/nc-shRNA or SW620/AF1q-shRNA cells were injected subcutaneously (1 × 10^6^ cells/mouse). Tumors were measured every five days, and tumor volume was calculated according to the following formula: tumor volume = (W + L)/2 × W × L × 0.5236. All mice were euthanized 40 days after injection. In the liver metastasis model, SW620/nc-shRNA or SW620/AF1q-shRNA cells were injected into the subcapsular region of the spleen (2 × 10^6^ cells/mouse). Liver metastatic lesions were examined 6 weeks later.

### 4.13. Statistical Analysis

Statistical analyses were performed using SPSS software version 19.0 (SPSS Inc., Chicago, IL, USA). Differences in AF1q expression between CRC tumor tissues and paired normal tissues were examined statistically using two-tailed, paired Student’s *t*-tests. The capacity for cell proliferation, apoptosis, wound-healing, migration, and invasion of the different CRC cell groups was compared using one-way ANOVA and Student’s *t*-tests. Survival curves were constructed using the Kaplan–Meier method; *p* < 0.05 was considered statistically significant.

## 5. Conclusions

In summary, we have demonstrated the oncogenic relevance of AF1q, and we have shown that up-regulation of AF1q expression is indicative of poor prognosis in CRC patients. Furthermore, our results are compatible with the conclusion that AF1q promotes CRC tumor progression and metastasis by facilitating EMT via AKT signaling. In the future, it will be interesting to continue this research in order to further prove our conclusion and to elucidate the intrinsic mechanisms by which AF1q regulates AKT phosphorylation.

## Figures and Tables

**Table 1:** The relationship between AF1q expression level and clinicopathologic variables in 96 CRC patients.

Clinicopathologic Parameters	AF1q Staining		
	Low (%) (*n* = 42)	High (%) (*n* = 54)	*p*-Value
Gender			
Male	22 (22.9)	31 (32.3)	0.623
Female	20 (20.8)	23 (24.0)	
Age (years)			
<50	6 (6.3)	7 (7.3)	0.851
≥50	36 (37.5)	47 (48.9)	
T			
T1, T2	9 (9.4)	11 (11.5)	0.899
T3, T4	33 (34.4)	43 (44.8)	
Tumor grade			
G1, G2	34 (35.4)	40 (41.7)	0.426
G3, G4	8 (8.3)	14 (14.6)	
Lymph node metastasis			
Negative	20 (32.3)	13 (13.5)	0.0159
Positive	22 (20.8)	41 (42.7)	
Distant metastasis			
Negative	40 (41.7)	45 (46.9)	0.135
Positive	2 (2.1)	9 (9.4)	
TNM Stage			
I, II	17 (17.7)	10 (10.4)	0.018
III, IV	25 (26.0)	44 (45.8)	

## Abbreviations

AF1q	ALL1-fused gene from chromosome 1q
EMT	Epithelial–mesenchymal transition
CRC	Colorectal cancer
AKT	Protein kinase B
